# Effects of Subsetting by Parent Materials on Prediction of Soil Organic Matter Content in a Hilly Area Using Vis–NIR Spectroscopy

**DOI:** 10.1371/journal.pone.0151536

**Published:** 2016-03-14

**Authors:** Shengxiang Xu, Xuezheng Shi, Meiyan Wang, Yongcun Zhao

**Affiliations:** 1 State Key Laboratory of Soil and Sustainable Agriculture, Institute of Soil Science, Chinese Academy of Sciences, Nanjing, China; 2 University of Chinese Academy of Sciences, Beijing, China; Old Dominion Univ., UNITED STATES

## Abstract

Assessment and monitoring of soil organic matter (SOM) quality are important for understanding SOM dynamics and developing management practices that will enhance and maintain the productivity of agricultural soils. Visible and near-infrared (Vis–NIR) diffuse reflectance spectroscopy (350–2500 nm) has received increasing attention over the recent decades as a promising technique for SOM analysis. While heterogeneity of sample sets is one critical factor that complicates the prediction of soil properties from Vis–NIR spectra, a spectral library representing the local soil diversity needs to be constructed. The study area, covering a surface of 927 km^2^ and located in Yujiang County of Jiangsu Province, is characterized by a hilly area with different soil parent materials (e.g., red sandstone, shale, Quaternary red clay, and river alluvium). In total, 232 topsoil (0–20 cm) samples were collected for SOM analysis and scanned with a Vis–NIR spectrometer in the laboratory. Reflectance data were related to surface SOM content by means of a partial least square regression (PLSR) method and several data pre-processing techniques, such as first and second derivatives with a smoothing filter. The performance of the PLSR model was tested under different combinations of calibration/validation sets (global and local calibrations stratified according to parent materials). The results showed that the models based on the global calibrations can only make approximate predictions for SOM content (RMSE (root mean squared error) = 4.23–4.69 g kg^−1^; *R*^2^ (coefficient of determination) = 0.80–0.84; RPD (ratio of standard deviation to RMSE) = 2.19–2.44; RPIQ (ratio of performance to inter-quartile distance) = 2.88–3.08). Under the local calibrations, the individual PLSR models for each parent material improved SOM predictions (RMSE = 2.55–3.49 g kg^−1^; *R*^2^ = 0.87–0.93; RPD = 2.67–3.12; RPIQ = 3.15–4.02). Among the four different parent materials, the largest *R*^2^ and the smallest RMSE were observed for the shale soils, which had the lowest coefficient of variation (CV) values for clay (18.95%), free iron oxides (15.93%), and pH (1.04%). This demonstrates the importance of a practical subsetting strategy for the continued improvement of SOM prediction with Vis–NIR spectroscopy.

## Introduction

Soil organic matter (SOM) is a key attribute of soil and environmental quality because it affects physical, chemical and biological functions, which in turn influence soil productivity [1]. Moreover, SOM contains large nutrient pools for crop growth and can serve as a source or a sink for atmospheric CO_2_ [2]. Quantitative assessment of SOM quality is therefore important for understanding SOM dynamics and provides valuable information for determining management practices that might maintain or increase SOM levels [3]. Typically, large numbers of samples must be collected and analyzed in order to capture the spatial and temporal variability of SOM [4]. Conventional methods may be expensive and require large amounts of labor and chemicals for performing these tasks [5].

Over the past three decades, visible and near-infrared diffuse reflectance spectroscopy (Vis–NIR) has been shown to be an effective alternative to conventional laboratory analysis, and can provide time and cost effective approaches for the prediction of various soil properties, including the SOM or SOC (the C within the SOM) content [6, 7, 8, 9,10, 11]. The basis for the Vis–NIR estimation of SOM content are its broad absorptions in the visible region (350–700 nm), due to chromophores and the darkness of humic acid, and the absorptions in the NIR region (700–2500 nm) from the overtones and combinations of fundamental vibrations due to stretching and bending of chemical bonds, such as O–H, C–H, and N–H [12, 13]. In fact, Vis–NIR spectroscopy is an indirect analytical method based on the development of multivariate statistical models, such as multiple linear regression [14], principal components regression (PCR) [15], partial least-squares regression (PLSR) [16], and non-parametric data mining techniques, including artificial neural networks and regression trees [9]. Among these methods, the PLSR is the most common technique used for prediction of soil properties when there are numerous predictor variables that are highly collinear [6, 13, 17, 18]. Meanwhile, different mathematical pre-processing transformations have been applied to raw reflectance spectra in order to improve estimation accuracy. The most commonly used are the first- and second-derivatives with a smoothing filter, which can remove baseline drift and background interference [19]. Depending on the quality of the raw spectral data, some studies employed the first derivative in SOM estimation [6, 15, 16, 20], while others preferred the second derivative [21, 22]. For example, prediction models based on the PLSR method and the first derivative have been developed by Chang et al. [15] and Dunn et al. [6] to estimate SOC content in the 400–2500 nm region.

Soil properties change because of natural (inherited) variation in the soil forming factors (i.e., climate, time, topography, vegetation, and parent materials) and human-made variation (e.g., tillage practices and fertilization) [23]. Of the five soil forming factors, much of the variation in soil mineralogy and also Vis–NIR spectra is likely to be explained by parent materials, which lead to differences in the type of clay formed [24; 25]. Previous studies have demonstrated that geographic regionality, such as changes in soil parent material, may affect prediction accuracies when using Vis–NIR spectroscopy [25, 26, 27, 28, 29]. Given that the relationship between soil properties and spectral data can be highly non-linear and spatially dependent [30], the main challenge limiting application of Vis–NIR technique for the prediction of soil properties is finding suitable data pretreatments and calibration strategies [15]. It difficult to construct a calibration that reflects the immense variation found in soils, even at a regional scale and so a large calibration does not guarantee accurate predictions [31]. However, spectral variation associated with soil properties can be locally stable [32]. Hence, one promising approach is to split the heterogeneous sample set into groups based on similar characteristics and to develop individual prediction models for each of these subsets [17, 33]. Previous researchers have investigated subsetting by characteristics such as parent materials [7, 24], soil types [10, 14, 34, 35], soil textures [36, 37], and spectral similarity [32] with varied results for their particular sample sets. For example, a work by Madari et al. [36] indicated that an excellent calibration performance, as expressed by the coefficient of determination (*R*^2^ = 0.87–0.96) for total carbon content, was obtained from 1135 soil samples from Brazil using sample subsets of different soil textural classes: very clayey, clayey, and medium texture. The same type of result was achieved by van Waes et al. [37] under laboratory conditions. They showed that dividing the samples into texture groups (clay, silt, and sand) improved the standard errors of prediction for agricultural grassland by 7%–16%. Stevens et al. [34] reported improved results with local calibrations stratified by soil types compared with global calibrations: the root mean square errors (RMSE) of prediction were 0.8–2.9 g C kg^−1^ and 5.3–6.2 g C kg^−1^, respectively. Likewise, as Vasques et al. [10] noted, the simple subsetting of 7120 samples in Florida by soil order improved the Vis–NIR SOC models in validation sets (RMSE = 0.33%–2.16%) relative to results that use the whole dataset (RMSE = 4.60%). The improvement observed with subsetting results from the fact that soil reflectance values are determined by constituents that vary greatly across heterogeneous samples. Subsetting matches spectra to a narrower set of data and improves accuracy. Nevertheless, other workers have shown some difficulties in gaining satisfactory absolute accuracy when including different parent materials [7]. Hence, it is essential to carry out a study concerning the Vis–NIR estimation of SOM content with samples derived from different parent materials when the local soil spectral libraries are unavailable.

In this context, a heterogeneous set of soil samples were included in our study that covered a relatively wide range of different parent materials and thus also a wide variation in SOM. The specific objectives are to: (i) investigate whether predictions from a PLSR model built only from a subset of samples that are similar with respect to parent materials will provide better predictions than a global model built from a set of all possible samples, and (ii) evaluate the effects of the raw vs. derivatives of spectral reflectance and the importance of wavelengths in the Vis–NIR for estimating SOM content.

## Materials and Methods

### Study area and soil sampling

The study area is Yujiang County (116°41′–117°09′E, 28°04′–28°37′N), located in the transition zone from the northeastern hilly area to the Poyang Lake Plain in Jiangxi Province, China, and covers an area of 927 km^2^. Permission to enter the area was issued by the Agricultural Bureau of Yujiang County. There is no endangered or protected species involved in the present study.

The study area is characterized by a warm climate, abundant heat and sunshine, plentiful rainfall, and a long frost-free period. Hills and plains cover 78% and 22% of the county, respectively. Low hills are the dominant landform, though high hills are found in the county’s north and south extremes. Arable agriculture accounts for more than 52% of land use over the area from which samples were collected. The dominant parent materials include red sandstone, shale, river alluvium, and Quaternary red clay [38]. The red sandstone is spread across large parts of the central and southern area, whereas the shale is centralized in the north hilly area. Soils developed from the four parent materials are predominantly red soil (Acrisols, WRB) and paddy soil (Anthrosols, WRB), which together account for over 90% of the county’s total area. Major crops include rice, peanut, and rape.

Soil sampling was undertaken across the study area at a density of one sample per 4 km^2^ in July and November of 2014 from croplands after crop harvest. A total of 232 geo-referenced sampling sites were chosen from different parent materials by simple random selection within each alternate kilometer square grid. Of the 232 sampling sites, 62, 51, 65, and 54 were taken from red sandstone, shale, river alluvium, and Quaternary red clay, respectively. At each site, a soil sample was composed of five sub-samples collected to a depth of 20 cm at random locations within a 10×10 m square centered on the geographical position of the site by a Dutch type soil auger. In the laboratory, samples were air-dried, passed through a 2-mm mesh sieve, and cleaned from visible plant residues. Each sample was then split into two sub-samples: one was used for the laboratory spectral measurements, while the other was used for the laboratory physicochemical analysis of four soil properties: SOM, soil pH, clay content, and free iron oxide content. The SOM content was determined by the Walkley–Black method [39]. The soil pH was measured in a 1:2.5 soil–water suspension using a glass electrode pH meter, and clay content was measured with the pipette method [40]. The free iron oxides were extracted with the dithionite citrate bicarbonate (DCB) method [41] and determined by atomic absorption spectroscopy.

As the frequency distribution of SOM was positively skewed, the SOM values were log-transformed to normalize the data for model developments. Estimated SOM contents from these models were back-transformed to original units (g kg^−1^) to assess model quality.

### Spectral measurement and pre-processing

**Fig 1:**
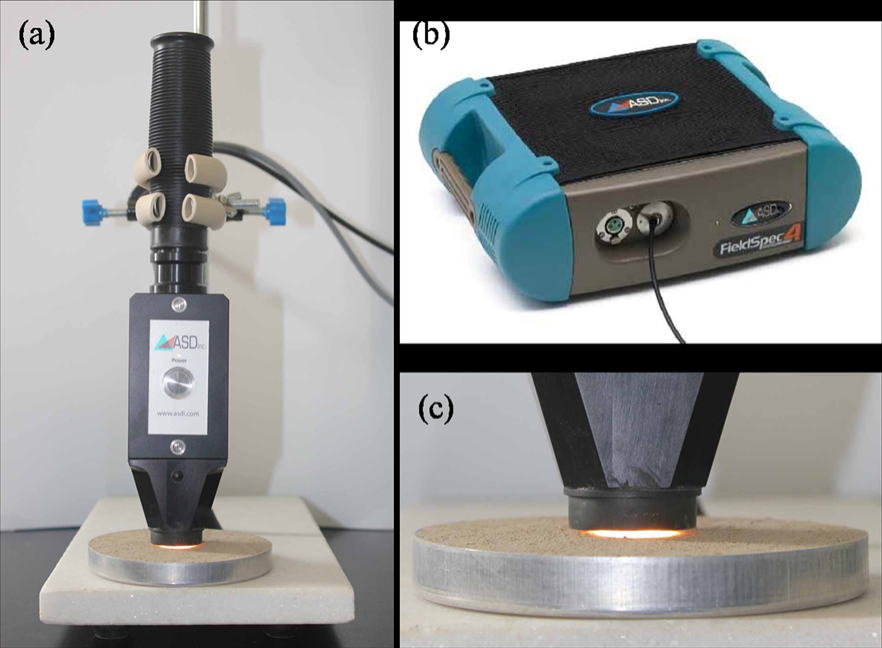
A soil probe developed to collect reflectance spectra of soils in Yujiang County of Jiangxi Province, China: (a) ASD contact probe, (b) ASD FieldSpec 4 spectroradiometer attached by a fiberoptic cable to the probe, and (c) soil sample.

To remove the effect of moisture, soil samples were oven-dried at 105°C for 24 h prior to measurements. Then, the diffuse reflectance spectra of the samples were measured in the laboratory in the Vis–NIR (350–2500 nm) range (Fig 1), with a spectral resolution of 3 nm (from 350 to 1000 nm) and 10 nm (from 1000 to 2500 nm), using a Fieldspec 4 spectroradiometer (Analytical Spectral Devices, Boulder, Colorado, USA). A high-intensity contact probe (also from Analytical Spectral Devices) with a built-in light source (6.5 W halogen lamp) and a measuring spot size of 10 mm, was used to acquire the soil spectra. To avoid disturbing soil surface, the probe was fixed to a burette stand with a clamp to avoid direct contact of the probe window with the soil sample and to provide a fixed distance of 3 mm between the probe and the soil sample (Fig 1C). Samples were placed in an aluminum dish (95-mm diameter, 15-mm height), and the soil surface was gently pressed before leveling with a spatula. This resulted in a smooth soil surface that ensured a maximum diffuse reflection and thus a good signal to noise ratio [42]. The sensor was calibrated with a Spectralon (Labsphere, North Sutton, NH) white reference once every 10 measurements. Each sample was scanned four times with a 90° rotation between 10 successive scans, and these forty readings were later averaged into one spectrum per sample. The output spectral resolution of the data is 1 nm along the whole spectrum. To eliminate noise at the edges of each spectrum, the raw spectra were first reduced to 380–2450 nm and then resampled in 5 nm increments across this range due to the highly collinear spectra, resulting in 415 bands for data analysis of 232 spectra (S1 File).

Before developing the SOM prediction models, several pre-processing techniques were applied, including the first and second derivatives (differentiation with second-order polynomial smoothing with a window width of 10 nm) using a Savitzky-Golay filter [43], a standard normal variate transform (SNV: [44]), and a multiplicative scatter correction (MSC: [19]). Overall, the three pretreatments (i.e., the second derivative, SNV, and MSC) were applied in order to obtain the best regression models but resulted in no improvement. Therefore, the results presented below are from the first derivative spectra.

### Multivariate calibration and validation

In this study, the PLSR method was used to correlate the spectral data with laboratory SOM measurements. PLSR is a predictive module technique used in spectroscopy and is closely related to PCR. However, unlike PCR, the PLSR algorithm selects orthogonal or latent factors that maximize the covariance between predictor (X spectra) and response variables (Y soil laboratory data). As a commonly used validation method [13], the leave-one-out cross-validation with as many as 10 factors was adopted in the PLSR model. The number of latent variables for a model was determined by examining a plot of leave-one-out cross-validation residual variance against the number of latent variables obtained from PLSR. The latent variable of the first minimum value of residual variance was selected [8]. Outliers were detected by using the residual sample variance plot after the PLSR. Samples individually located far from the zero line of residual variance were considered to be outliers and were excluded from the analysis [45]. More detailed information about the PLSR technique can be found in [46].

The stability of the prediction models was verified by test-set validation with a 3:1 ratio of calibration and validation samples. Two types of combinations of calibration/validation sets were compared to study the effect of sample set heterogeneity on Vis–NIR prediction. First, a “global” calibration/validation set containing spectral data for the full sample set area was created (called the global PLSR). Secondly, a series of “local” calibration/validation sets regrouped by soil parent materials were constructed to produce an individual model for each type separately (called the local PLSR). This strategy has been adopted because using spectral data from areas with heterogeneous soil parent material or soil type is known to diminish the predictive ability of Vis–NIR spectroscopy [17]. For each type of combination, the soil samples were divided into calibration and validation data sets by using a stepwise partitioning scheme, whereby validation data comprised every fourth observation after sorting the SOM content of all samples in an ascending order [18]. The independent validation sets were used to test the accuracy and robustness of the calibration models developed using spectra that were not used in the PLSR cross-validation.

The prediction accuracy of the model for the calibration and validation datasets was evaluated through parameters such as *R*^2^, RMSE, the ratio of prediction to deviation (RPD), and ratio of performance to inter-quartile distance (RPIQ) [47, 48]. The equations describing the statistics employed are as follows:

$$R^{2}=\sum_{i=1}^{N}{\left({\hat{y}}_{i}-{\bar{y}}_{i}\right)}^{2}/{\left(y_{i}-{\bar{y}}_{i}\right)}^{2}$$ (1)

$$RMSE=\sqrt{\sum_{i=1}^{N}{\left({\hat{y}}_{i}-y_{i}\right)}^{2}/N}$$ (2)

RPD=SD/RMSE (3)

RPIQ=IQ/RMSE (4)

where $\hat{y}$ is the predicted value, *y* is the observed value, $\bar{y}$ is the mean of observed values, *N* is the number of data points, *SD* is the standard deviation of the observed values, and *IQ* is the inter-quartile distance of the measured values. According to Zornoza et al. [47], a RPD < 2 is considered insufficient for applications, whereas a value for RPD between 2 and 2.5 makes approximate quantitative predictions possible. For RPD values between 2.5 and 3.0 and above 3.0, the prediction is classified as good or excellent, respectively. Generally, a good model prediction would have large values of *R*^2^, RPD, and RPIQ, and a small value of RMSE.

To determine the significant wavelengths used in calibrations, the PLSR models were also assessed with variable importance in projection (VIP) as well as PLS regression coefficients (called b-coefficients) [46, 48]. The VIP was calculated using:

$$VIP_{k}\left(a\right)=K\sum_{a}w_{ak}^{2}\left(SSY_{a}/SSY_{t}\right)$$ (5)

where *VIP*_*k*_(*a*) is the importance of the *kth* predictor variable based on a model with *a* factors, *w*_*ak*_ is the corresponding loading weight of the kth variable in the ath PLSR factor, *SSY*_*a*_ is the explained sum of squares of y by a PLSR model with a factors, *SSY*_*t*_ is the total sum of squares of y, and *K* is the total number of predictor variables. Thresholds were introduced for the determination of important wavebands [46]. The thresholds for the VIP were set to 1 and thresholds for the b-coefficients were based on their standard deviations [5, 49]. The wavelength was considered to be important if both the values (VIP score and b-coefficient) exceeded the thresholds.

### Data analysis

All data pretreatments and PLSR calibrations were performed with the Unscrambler 9.7 software (Camo Inc., Oslo, Norway). No samples were considered outliers or excluded from the analyses. In addition to using the PLSR models, Pearson correlations were computed to study the relationships between SOM content and measured reflectance for each wavelength of the entire spectral range of 380–2450 nm. This analysis was carried out using SPSS version 18.0 for Windows (SPSS Inc., Chicago, IL).

## Results and Discussion

### Descriptive statistics

A summary of the statistics for laboratory SOM data analyzed with respect to the whole dataset, calibration set, and validation set are given in Table 1. Considering the whole dataset, the SOM contents varied from 10.59 up to 58.95 g kg^−1^ with a mean of 30.23 g kg^−1^ and differed between parent material types. For instance, soils derived from Shale contained, on average, more than 42.2% of the SOM content observed in the Quaternary red clay. Except for the shale samples, the SOM content also showed also a relatively high variability within the same parent material. The whole SOM contents had a positively skewed distribution (skewness = 0.23). In the calibration set, the SOM content ranged from 10.59 to 56.27 g kg^−1^ with a standard deviation (SD) of 9.94 g kg^−1^. A similar range of SOM values (11.82–58.95 g kg^−1^) with a SD of 10.30 g kg^−1^ was presented in the validation set. The fact that both calibration and validation sets have similar descriptive statistics indicates that stepwise selection followed by SOM stratification can be used to represent the main variability of soil samples.

**Table 1:** Statistical characteristics of the organic matter content of soil samples developed from different parent materials in Yujiang County of Jiangxi Province, China.

	N	SOM (g kg^-1^)				CV (%)	Skewness
		Minimum	Maximum	Mean	SD		
Original dataset							
Red sandstone	62	10.59	53.57	28.81	10.07	34.95	0.46
Shale	51	17.90	58.95	37.93	9.02	23.78	0.14
Quaternary red clay	54	11.45	42.51	26.67	9.10	34.12	-0.21
River alluvium	65	13.68	50.82	28.49	8.36	29.34	0.29
Full	232	10.59	58.95	30.23	10.01	33.11	0.23
Calibration							
Red sandstone	47	10.59	53.57	28.71	10.30	35.88	0.45
Shale	39	17.90	58.95	37.90	9.44	24.91	0.12
Quaternary red clay	41	11.45	42.51	26.56	9.25	34.83	-0.19
River alluvium	49	13.68	50.82	28.33	8.45	29.83	0.30
Full	174	10.59	56.27	30.11	9.94	33.01	0.18
Validation							
Red sandstone	15	13.81	49.94	29.10	9.69	33.30	0.61
Shale	12	25.39	52.86	38.02	7.89	20.75	0.38
Quaternary red clay	13	12.90	39.99	27.04	8.97	33.17	-0.28
River alluvium	16	15.89	45.69	28.97	8.32	28.72	0.34
Full	58	11.82	58.95	30.57	10.30	33.69	0.36

Note that: N, number of samples; SOM, soil organic matter; SD, standard deviation of SOM content; CV, coefficient of variation of SOM content.

### Soil spectral characteristics

**Fig 2:**
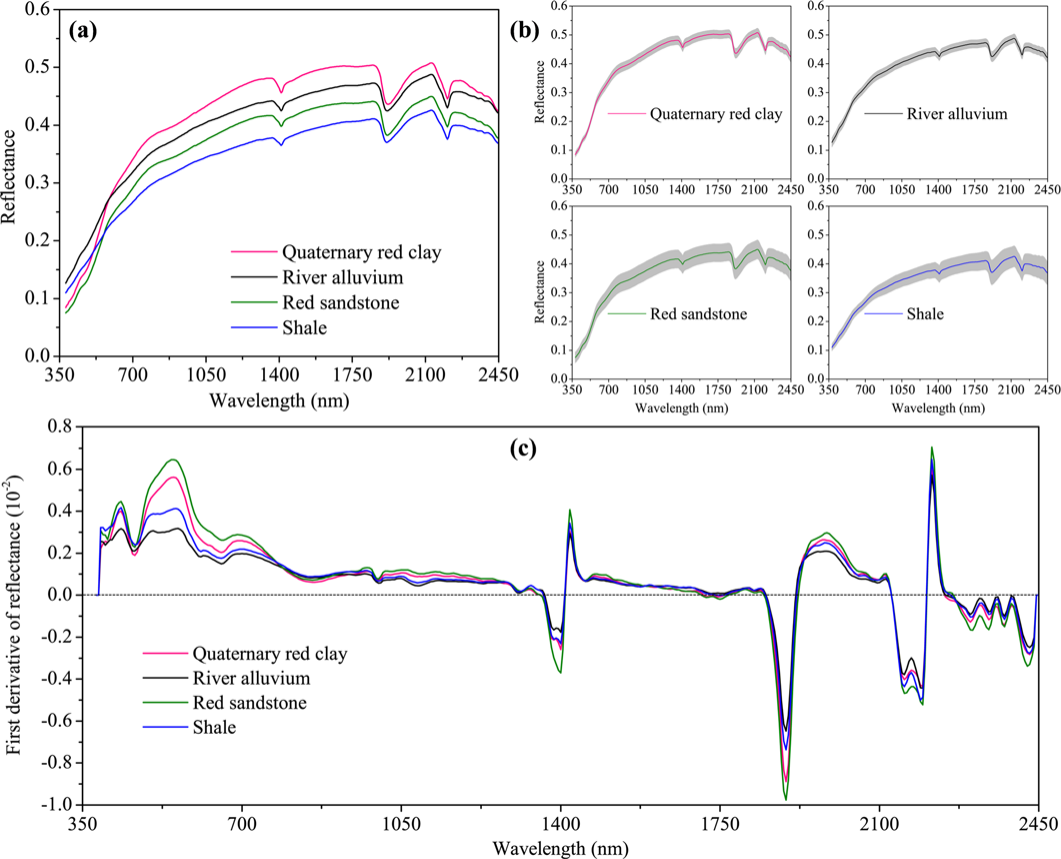
Average spectral curves of (a) the raw reflectance, (b) their corresponding standard deviation values (shaded regions), and (c) the first derivative of reflectance for the soil samples developed from different parent materials in Yujiang County of Jiangxi Province, China.

The mean Vis–NIR spectra of cropland soils developed from different parent materials (Fig 2A) and their respective standard deviations have basic shapes similar to those observed by other studies [7, 50]. In the 380–760 nm range, the reflectance profiles showed a rising trend and shifted quickly toward the long-waveband direction. In the 850–2350 nm range, the reflectance spectra changed gently. All soil reflectance spectra exhibited prominent absorption features at approximately 1400, 1900 and 2200 nm, which are strongly associated with clay minerals, for example the OH features of free water at 1400 and 1900 nm, and clay lattice OH features at 1400 and 2200 nm [50, 51]. Absorption peaks were generally enhanced in the first derivative graphs relative to the raw reflectance graphs (Fig 2C).

In general, increasing SOM concentration would lower reflectance magnitude across the whole Vis–NIR spectrum [15, 50]. Average spectral curves for the raw reflectance decreased from Quaternary red clay, river alluvium, red sandstone, and shale (Fig 2A). The observed decrease in reflectance may be partly related to mineralogy but also to differences in SOM content. The parent material has an indirect effect on soil reflectance by influencing soil mineralogy and texture [52]. Soils originating from the Quaternary red clay had low organic matter content (on average 26.67 g kg^-1^) and had a reddish color. By contrast, soils from shale had higher organic matter content (on average 37.93 g kg^-1^) and appeared brownish in color. The Yujiang’s cropland soils in South China had an intermediate SOM content and varied iron oxide content in different parent materials (Table 2). Water and iron oxides are considered, along with organic matter, to be the main soil chromophores [50]. Iron oxides adsorb strongly in the ultraviolet and blue spectral regions, but are strongly reflecting in the red and infrared regions (800–1000 nm). The spectral region of 690–930 nm that is influenced by SOM and iron oxide is the recombination region [13, 53], and the presence of SOM tended to subdue the iron oxide reflectance features in the 600–750 nm range [50]. From Fig 2B it can be observed that several absorption features occurred in the regions at approximately 430, 560, and 850 nm, which is consistent with other research findings [3, 54]. Thus, the spectral variability induced by these two constituents is likely to be reduced when considering the different soil parent materials separately.

**Table 2:** Statistical characteristics of soil physicochemical variables for soil samples developed from different parent materials in Yujiang County of Jiangxi Province, China.

Parent material	N	pH			Clay (%)			Free iron oxides (g kg^-1^)		
		Range	Mean	CV	Range	Mean	CV	Range	Mean	CV
Red sandstone	62	4.52–5.13	4.86	2.88	13.64–29.00	17.63	21.55	2.37–22.57	8.64	57.80
Shale	51	4.75–4.96	4.80	1.04	15.56–34.36	25.06	18.95	13.61–23.76	19.00	15.93
Quaternary red clay	54	4.66–5.08	4.87	1.85	15.48–26.98	19.67	20.62	6.63–19.56	11.57	24.98
River alluvium	65	4.53–5.36	4.83	3.73	10.52–20.04	15.10	19.01	1.62–17.57	5.62	54.73
Full	232	4.52–5.36	4.84	2.69	10.52–34.36	19.03	26.75	1.62–23.76	10.75	58.60

Note that: N, number of samples; CV, coefficient of variation of soil variables (%).

### Correlation of SOM and Vis–NIR spectra

**Fig 3:**
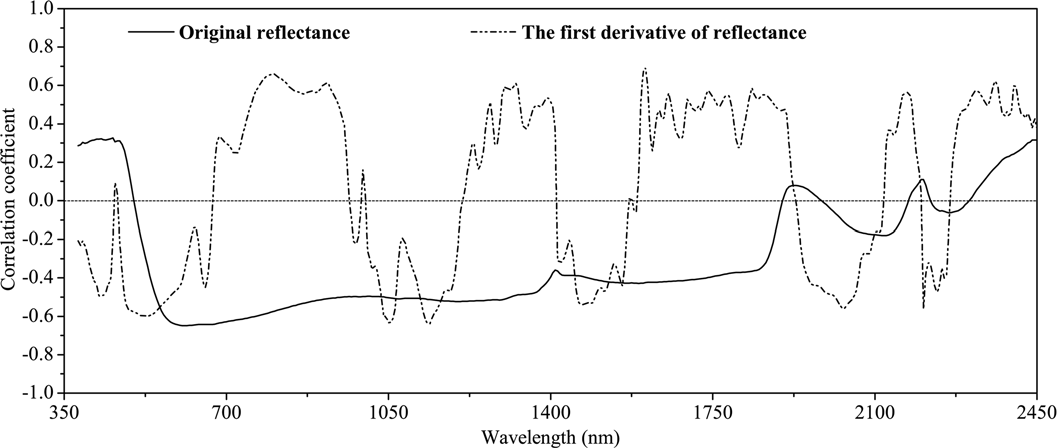
Correlation of the organic matter content of the soil samples in Yujiang County of Jiangxi Province, China with the original reflectance and the first derivative of the original reflectance (n = 232) at different wavelengths.

The importance of different wavelength regions can be assessed using correlation plots. In our study, the correlation coefficients between SOM and reflectance spectra showed both positive and negative peaks across the spectrum (Fig 3). The SOM content was well correlated with the original reflectance and the first derivative treatments of the absorbance. For the original reflectance, the bands at approximately 560–850 nm had relatively high correlation coefficients (r_max_ = –0.65). For the first derivative spectra, the bands at approximately 485–580 nm (r_max_ = –0.60), 760–940 nm (r_max_ = 0.66), 1035–1165 nm (r_max_ = –0.63), 1270–1400 nm (r_max_ = 0.61), 1600–1900 nm (r_max_ = 0.69), 2020–2055 nm (r_max_ = –0.56), 2160–2180 nm (r_max_ = 0.56), and 2310–2370 nm (r_max_ = 0.62) were highly correlated with SOM content. The bands at 1340–1380 nm are usually associated with the carbon–hydrogen (C–H) bonds, while the bands at 1860–1900 nm are related to amide nitrogen–hydrogen (N–H) and O–H bonds [50]. Although the best correlation coefficients were generally observed in the NIR region, preliminary investigations did not show a significant improvement in prediction accuracy when only the NIR region was used instead of the whole spectrum [20]. Hence, for the purpose of calibrating soil properties to spectral characteristics, it is preferable to use information over the entire spectrum, rather than attempting to interpret individual absorption features. Soil spectra result from overlapping absorption features of many organic and inorganic components, and thus subtle differences in spectral shape may provide valuable information about soil properties.

### SOM prediction by PLSR analysis

**Fig 4:**
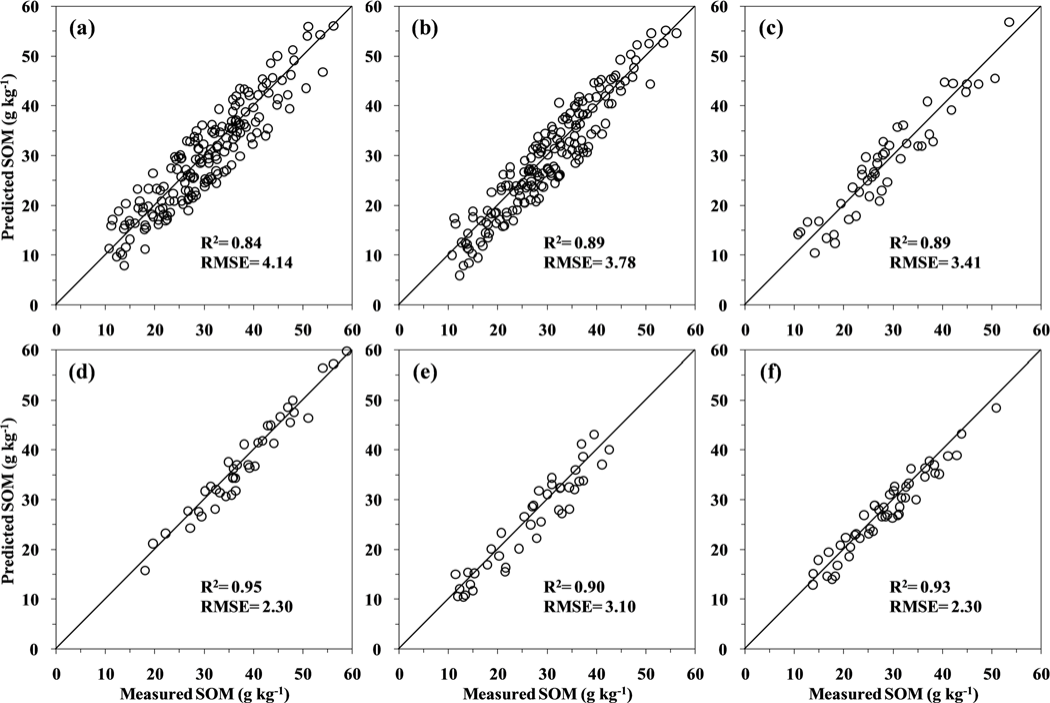
Plots of the measured versus predicted SOM content in the calibration sets obtained via the local PLSR models for soils in Yujiang County of Jiangxi Province, China: (a) the original spectra and (b) the first derivative spectra, and the global PLSR models for (c) the red sandstone, (d) the shale, (e) the Quaternary red clay, and (f) the river alluvium. The 1:1 line is indicated on each figure.

**Fig 5:**
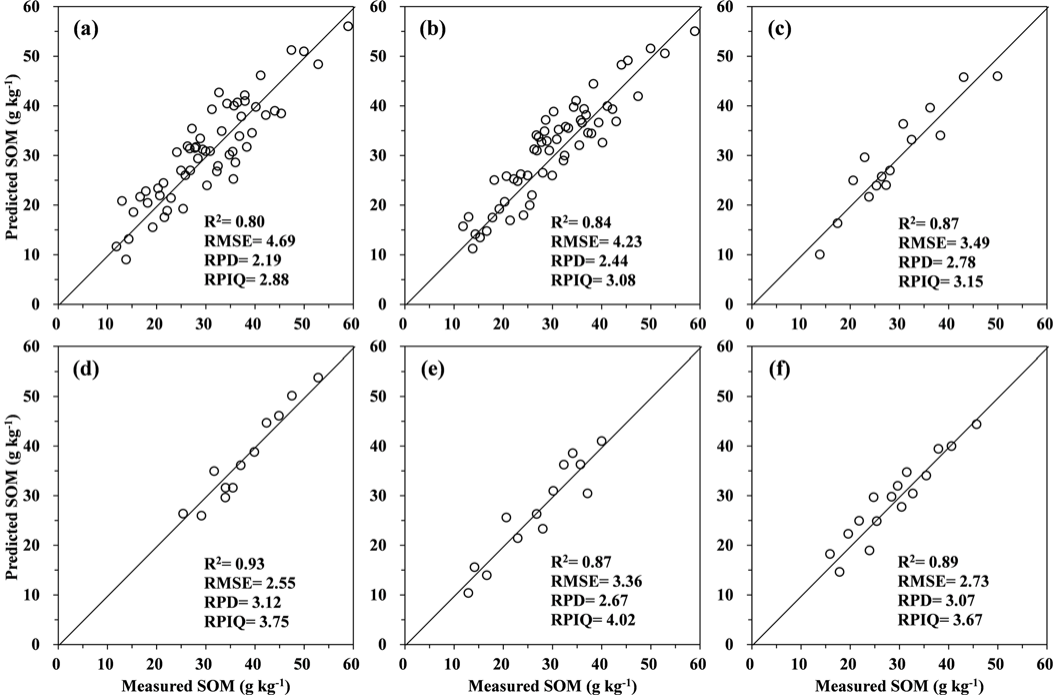
Plots of the measured versus predicted SOM content in the validation sets obtained via the local PLSR models for soils in Yujiang County of Jiangxi Province, China: (a) the original spectra and (b) the first derivative spectra, and the global PLSR models for (c) the red sandstone, (d) the shale, (e) the Quaternary red clay, and (f) the river alluvium. The 1:1 line is indicated on each figure.

The best pretreatments of spectral data for each PLSR calibration technique were identified based on the highest RPD values. Model performance statistics are summarized in Figs 4 and 5, which plot the laboratory measured and predicted SOM concentrations using PLSR analysis for the calibration and validation data sets, respectively. The *R*^2^ values in the validation set were lower and RMSE values were higher than the corresponding values in the calibration set, but statistical performance was not much different. The cross-validation approach gave over-optimistic results in terms of SOM predictions for new unknown samples. It is uncommon to obtain similar prediction levels when independent samples are used for validation [55], even if the data sets were both from the same spatial domain. Validation tests using samples that were not involved in the calibration test were thus necessary.

When considering global calibrations (Fig 5A and 5B), the first derivative transformation was generally more accurate and had higher performance values (*R*^2^ = 0.84, RPD = 2.44, and RPIQ = 3.08) than the original spectral prediction (*R*^2^ = 0.80, RPD = 2.19, and RPIQ = 2.88) based on the validation set. The RPD values for these two methods were both less than 2.5 which means that the models can just make approximate quantitative predictions possible. This finding was in line with those of the other studies [8], in which the first derivative worked the best. Based on these results, the remaining local calibration models were performed by using the first derivative of the Vis–NIR spectra for the four soil parent materials. Fig 5 also shows that the two local PLSR models improved the prediction ability for the red sandstone (*R*^2^ = 0.87, RPD = 2.78, and RPIQ = 3.15) and the Quaternary red clay soils (*R*^2^ = 0.87, RPD = 2.67, and RPIQ = 4.02), indicating that they were good prediction models. Excellent predictions were obtained from the shale and river alluvium soils, with RPD values greater than 3.0 (Fig 5D and 5F). The relatively poor results from the global calibrations may be due to the heterogeneity of the sample set, as optimal calibration requires limited (but sufficient) set heterogeneity [56]. The weak relationship between the SOM and its reflectance when soil samples are collected from large geographic areas has been attributed to probable parent material influences on soil mineral reflectance [57]. This would suggest that the local PLSR model could be used to predict SOM values for similar soil parent materials in the region, even for samples pre-screened for spectral similarity [7]. It is supported by the improvements presented by Sankey et al. [31] when predicting SOC at one site using a subset of calcareous soil samples compared to using the whole library.

When applying local calibrations, the relatively similar RMSE values in the calibration (2.30–3.41 g kg^−1^) and validation (2.55–3.49 g kg^−1^) stages ensured that the extracted models were stable and reliable for further use. The accuracy of SOM predictions with independent validation was roughly comparable to other published studies [4, 8, 58, 59]. By using the first derivative Vis–NIR PLSR modeling and completely random 30% test sets, Brown et al. [8] obtained validation RMSE values of 1.09–1.27 g kg^−1^ for SOC in six sites with similar soils across three counties in north central Montana. Wetterlind et al. [58] reported a farm-scale calibration model for SOM using 25 soil samples only, and achieved a good prediction (*R*^2^ = 0.89, RMSE = 4.70 g kg^−1^, and RPD = 3.0) at Hacksta in southern Sweden. Over a dataset of 152 samples with variability in nine soil types, O'Rourke and Holden [60] reached a RMSE of 4.46 g kg^−1^ and a RPD of 2.49 for SOM validation model by applying the first derivative with Savitzky–Golay smoothing technique. It was also reported by Tian et al. [59] that the PLSR calibration model with the first derivative spectra for estimating SOM gave a better performance (*R*^2^ = 0.91, RMSE = 3.11 g kg^−1^, and RPD = 3.48) than the original spectral reflectance (*R*^2^ = 0.84, RMSE = 4.24 g kg^−1^, and RPD = 2.55), which was conducted for five different soil types originating from seven eco-climatic zones in middle and eastern China. The above comparisons suggest that the results of the present study were very satisfactory for heterogeneous soil sample sets in a hilly area.

In spite of the fact that the global models have larger sample concentration ranges than all the individual parent materials, the greater soil variability (e.g., clay, free iron oxides, and parent materials) of the mixed sample set has resulted in smaller *R*^2^ and RPD values. Because the soils for the study area were developed under similar varieties and distribution of vegetation, land-use, and pedogenic factors, the variation of soil properties could be attributed to the different geological genesis and parent materials, as reflected by the Vis–NIR spectra as well as physicochemical analysis. As a consequence of the spatial proximity and similar parent materials, soil samples from the same zone are more likely to exhibit less variation in soil properties and facilitate more accurate predictions [30]. Our analysis has shown that there are substantial differences in the performance of PLSR models for SOM prediction based on Vis–NIR for soils developed over different parent material types across the study area. Regarding the individual parent materials, the quality of validation statistics for SOM increased as set homogeneity of parent materials (expressed by CV of soil physicochemical properties) increased. Among the four different parent materials, the largest *R*^2^, RPD, and the smallest RMSE were observed in the validation dataset for the shale soils, which had the lowest CV values for clay (18.95%), free iron oxides (15.93%), and pH (1.04%). Similarly, Peng et al. [28] demonstrated that the accuracy of the models from the subsets depended on the soil parent material by using the Danish soil spectral libraries to predict SOC at the field scale. It is noteworthy that the interest of Vis–NIR spectroscopy for quantifying soil properties depends on the proportion of samples used for calibration; it also depends on the stability of predictions when different calibration sample selections are carried out. As seen from this study, the *R*^2^ or RPD values of the PLSR models were higher compared to previous studies [4, 6, 7, 8, 10, 32, 45], which might be due to the small samples size, large range in SOM, and the sample dividing method of stepwise selection.

**Fig 6:**
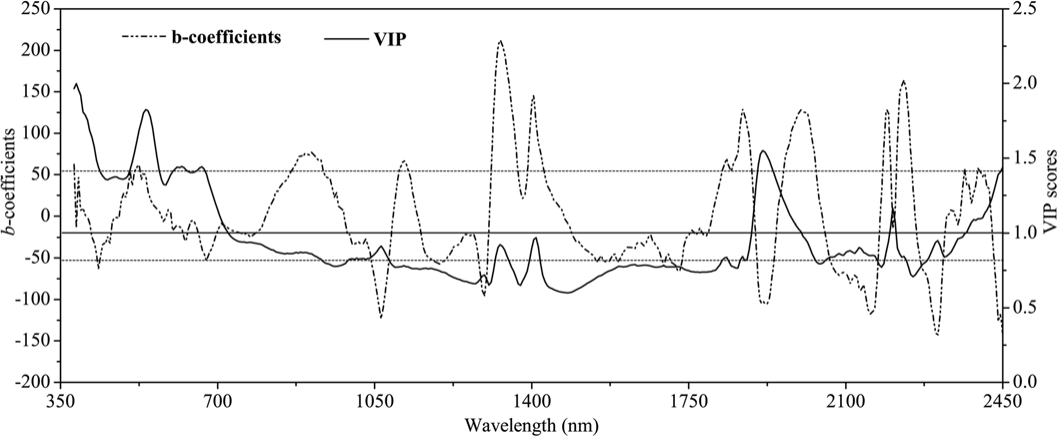
Plots of variable importance projection (VIP) scores (solid curves) and *b*-coefficients (dashed curves) associated with the PLSR cross-validation model for organic matter content of the soil samples in Yujiang County of Jiangxi Province, China. The threshold for the VIP was set to 1 (solid horizontal line) and the thresholds for the *b*-coefficients were based on their standard deviation (σ = 52.4) (dashed horizontal lines).

To reveal possible coherence in the selection of important variables for predicting SOM from PLSR calibrations, the VIP scores and b-coefficients (Fig 6) are plotted on an average soil spectrum. The important variables with VIP > 1 mainly lay in the regions of 380–720 nm and approximately 1900 nm, with some in the region of 2370–2450 nm. In terms of b-coefficients, the wavelengths near 435, 530, 900, 1060, 1300, 1330, 1400, 1730, 1870, 1900, 2000, 2160, 2230, 2300, and 2440 nm were identified as important wavelengths for PLSR modeling. This finding reinforced the evidence given by other authors [9, 11, 42, 57, 61], who had also reported association of important wavelengths identified by PLSR analysis for predicting SOM or SOC. For example, Henderson et al. [57] found that short-wave infrared bands (1955–1965, 2215, 2265, 2285–2295, and 2315–2495 nm) gave high correlation with SOC content (*R*^2^ > 0.96). Additionally, Lee et al. [61] reported 450–550, 900, 1400, and 1775–2200 nm as important wavelengths for SOC. Vasques et al. [11] identified wavelengths 400, 1000, 1400, 1900, 2100, and 2200 nm as important for different forms of carbon. Moreover, some other researchers found that wavelengths greater than 1200 nm are associated closely with SOM [14, 57, 62]. These bands have the advantage of being uncorrelated with iron oxide and therefore may demonstrate a higher predictive capacity for different parent materials [57]. The latter results agreed with the findings of the present study, as the most successful SOM prediction model (with local PLSR calibrations) used more than 15 spectral variables at wavelengths greater than 1200 nm (Fig 6). On the other hand, the complexity of both SOM chemistry and the SOM spectral response make it difficult to assign absorption features to specific SOM functional groups, resulting in a highly variable use of wavelengths in different predictive models [63]. This variability is the main reason that researchers often tend only to develop local calibration models for each field in which they make measurements with Vis–NIR spectroscopy [42].

## Conclusions

Based on comprehensive analysis of the relationship between SOM content and corresponding reflectance spectra in the four different parent materials from a hilly area in Yujiang County, we have provided the basis for future study of sample subsetting for large datasets based on parent material types. This particular investigation of subsetting for SOM prediction had varied results with a sample set containing 232 soils. The different subset models created based on parent material types showed improvement across all parameters (i.e., *R*^2^, RMSE, RPD, and RPIQ) compared to the full sample set. The best prediction for SOM was obtained from the shale parent material with validation *R*^2^, RMSE, RPD, and RPIQ values of 0.93, 2.55, 3.12, and 3.75, respectively. The lower RMSE values in validations (< 3.49 g kg^−1^) from these local models could be very helpful for monitoring of small changes in SOM content. Moreover, although not addressed in this paper, there are indications that other important soil properties such as clay content, pH value, cation exchange capacity, and nitrogen content can simultaneously be analyzed using Vis–NIR spectroscopy and local PLSR models. The above results are quite promising; however, they have the drawback that parent material information is not always available for a new soil sample. In future studies, the practical implementation of the subsetting strategy is done either by including readily available soil covariates (e.g. mineralogy, texture, and iron oxide content) in the spectroscopic modeling or by building a spectral library for all kinds of parent materials in a region. In addition, the developed models with different calibration/validation groupings need to be tested further across a wider range of soils characterized by similar parent materials to confirm their wider applicability.

## Supporting Information

S1 File Laboratory measured Vis–NIR spectra of 232 samples. To every fifth wavelength was retained to reduce the size of the file.
(XLSX)
